# On Visually-Grounded Reference Production: Testing the Effects of Perceptual Grouping and 2D/3D Presentation Mode

**DOI:** 10.3389/fpsyg.2019.02247

**Published:** 2019-10-01

**Authors:** Ruud Koolen

**Affiliations:** Tilburg Center for Cognition and Communication, Tilburg University, Tilburg, Netherlands

**Keywords:** reference production, perceptual grouping, overspecification, visual scene perception, 2D and 3D visual processing, eye tracking

## Abstract

When referring to a target object in a visual scene, speakers are assumed to consider certain distractor objects to be more relevant than others. The current research predicts that the way in which speakers come to a set of relevant distractors depends on how they perceive the distance between the objects in the scene. It reports on the results of two language production experiments, in which participants referred to target objects in photo-realistic visual scenes. Experiment 1 manipulated three factors that were expected to affect perceived distractor distance: two manipulations of perceptual grouping (region of space and type similarity), and one of presentation mode (2D vs. 3D). In line with most previous research on visually-grounded reference production, an offline measure of visual attention was taken here: the occurrence of overspecification with color. The results showed effects of region of space and type similarity on overspecification, suggesting that distractors that are perceived as being in the same group as the target are more often considered relevant distractors than distractors in a different group. Experiment 2 verified this suggestion with a direct measure of visual attention, eye tracking, and added a third manipulation of grouping: color similarity. For region of space in particular, the eye movements data indeed showed patterns in the expected direction: distractors within the same region as the target were fixated more often, and longer, than distractors in a different region. Color similarity was found to affect overspecification with color, but not gaze duration or the number of distractor fixations. Also the expected effects of presentation mode (2D vs. 3D) were not convincingly borne out by the data. Taken together, these results provide direct evidence for the close link between scene perception and language production, and indicate that perceptual grouping principles can guide speakers in determining the distractor set during reference production.

## Introduction

**Figure 1:**
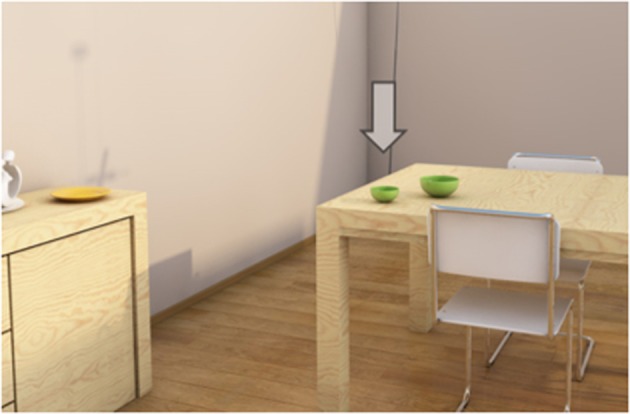
An example visual scene.

Definite object descriptions (such as “*the green bowl”* or “*the large green bowl”*) are an important part of everyday communication, where speakers often produce them to identify objects in the physical world around them. To serve this identification goal, descriptions should be unambiguous, and must contain a set of attributes that jointly exclude the *distractor objects* with which the listener might confuse the *target object* that is being referred to. For example, imagine that a speaker wants to describe the object that is pointed at with an arrow in Figure 1.

Solving the referential task here requires *content selection*: the speaker must decide on the attributes that she includes to distinguish the bowl from any distractor object that is present in the scene (such as the other bowl, the plate, and the chairs). This notion of content selection does not only reflect human referential behavior, but is also at the heart of computational models for referring expression generation. Such models, most notably the Incremental Algorithm (Dale and Reiter, 1995), typically seek attributes with which a target object can be distinguished from its surrounding distractors, aiming to collect a set of attributes with which any distractor that is present in the scene is ruled out (Van Deemter et al., 2012).

What would a description of the target object in Figure 1 look like? The target's type is probably mentioned because it is necessary for a proper noun phrase (Levelt, 1989). Also size is likely to be included, to rule out the large bowl. What else? The speaker may also add color, following the general preference to mention this attribute (e.g., Pechmann, 1989; Arts, 2004; Belke, 2006), or because the speaker is triggered by the different colors of the objects present in the visual scene (Koolen et al., 2013). In the strict sense, adding color would cause the description to be *overspecified* (Pechmann, 1989), since the color attribute is not required for unique identification: mentioning type and size (“*the small bowl”*) would rule out all possible distractors in the current visual scene. Please note that in line with most of the previous papers on referential overspecification (starting with Pechmann, 1989), the current paper applies such a strict definition of overspecification, where references are overspecified when they contain at least one *redundant* adjective that is not necessary for unique target identification. However, I am aware that overspecified referring expressions are sometimes pragmatically felicitous (e.g., Rubio-Fernández, 2016).

In the current study, overspecification with color is taken as one of the key dependent variables, providing an offline measure of visual attention, in line with various recent papers on visually-grounded reference production (e.g., Pechmann, 1989; Koolen et al., 2013; Rubio-Fernández, 2016; Viethen et al., 2017). Those papers show that overspecification is often elicited by color variation, which is a finding that seems to be reflected in the vision literature. In fact, a reference production task such as the one described here can be seen as some form of guided search (Gatt et al., 2017), where the speaker has a target object in focus and compares its properties to the properties of the distractors. Within that process, color is typically one of the salient features: color differences usually “pop out” of the scene (e.g., Treisman and Gelade, 1980), and are therefore central to early visual processing (e.g., Itti and Koch, 2001; Wolfe and Horowitz, 2004; Wolfe, 2010). As guided search gets easier as the color variation between target and distractors gets higher (Bauer et al., 1996), it is in turn plausible to assume that speakers are triggered by color variation when referring to a target whose color is clearly different from the color of its distractors (such as in Figure 1), and that this may result in overspecification.

If color variation can trigger a speaker to use a redundant color attribute, this implies that the distractors in a visual scene largely determine the process of content selection. For the case of Figure 1, it might be the case that the speaker would only add color if she regarded *all* objects in the scene as relevant distractors, producing “*the small green bowl”* as her final description. However, there are reasons to believe that speakers generally ignore some distractors (Koolen et al., 2016), and consider certain distractors to be more relevant than others. What makes an object a relevant distractor? The current paper explores the impact of two factors that may affect how speakers perceive the distance between the target and its surrounding objects, and, in turn, tests how they influence content selection for referring expressions. The factors that are manipulated are *perceptual grouping* (in various appearances) and *presentation mode* (2D/3D).

The effects of perceptual grouping and presentation mode are investigated in two reference production experiments, which are different in the dependent variables that are measured. Experiment 1 investigates overspecification with color as a function of both grouping and presentation mode. Overspecification is there taken as an indicator of perceived distractor distance. Experiment 2 focuses solely on perceptual grouping, for which it adds a new manipulation, and a direct measure of visual attention: eye movement data. This way, the second experiment applies a combination of offline (overspecification with color) and online (eye tracking) measures of visual scene perception, and tests how different manipulations of perceptual grouping cause some distractor objects in the visual scene to be fixated, and others to be ignored. Below, I first discuss the expectations for the effects of perceptual grouping and presentation mode on overspecification with color in more detail, followed by a description of Experiment 1, including results and interim discussion. The expectations for the various manipulations of grouping on eye movements, as well as the added value of eye tracking measures in language production research, follow as an introduction to Experiment 2, after the description of Experiment 1.

## Perceptual Grouping

The starting point of this study is the assumption that in a reference production task, speakers do not regard all objects in a visual scene to be relevant distractors, but rather rely on a subset of distractor objects. More specifically, speakers are expected to only consider the distractors that are in their focus of attention (Beun and Cremers, 1998). One can think of various factors that determine whether an object is perceived or not, such as its physical distance to the target (i.e., proximity). Given that proximity predicts that only objects that are close to the target are in the speaker's focus of attention, it can restrict the distractor set that is relied on, and, in turn, affect the content of the resulting object description. Regarding the latter, Clarke et al. (2013a) found that in heavily cluttered scenes, certain distractors are less likely to be included as a landmark in a referring expression as they are farther away from the target. However, in a recent study by Koolen et al. (2016), systematic effects of proximity on reference production were not borne out by the data.

Proximity is only one of the classical Gestalt laws of *perceptual grouping* that were originally introduced by Wertheimer (1923), next to similarity, closure, continuation, and pragnanz. These laws are all principles of perceptual organization and serve as heuristics: they are mental shortcuts for how we perceive the visual environment (Wagemans et al., 2012), and for how we create meaningful groups of the objects that we see around us (Thórisson, 1994). As such, perceptual grouping principles affect visual scene inspection. One of the dominant perspectives in the vision literature is that visual attention is often object-based (Egly et al., 1994). Object-based attention holds that viewers' attention is often directed to the “objects” in a scene rather than to the locations that may be prominent. These “objects” are in fact the perceptual groups of entities or units that are formed by the viewer, pre-attentively, based on perceptual grouping principles such as the ones mentioned above. By manipulating various grouping principles, the current paper investigates how object-based attention can be guided, and, in turn, affects reference production.

Perceptual grouping has been investigated thoroughly in the field of visual search, where scholars have studied how the extent to which a certain visual scene affords the formation of groups of objects leads to greater target search efficiency. For example, based on some influential early work showing that the search time for a target in a visual scene increases with the number of features that need to be processed (e.g., Treisman and Gelade, 1980; Nakayama and Silverman, 1986), Nordfang and Wolfe (2014) report a series of experiments on the role of grouping in target identification tasks. They created visual scenes of abstract objects that could vary on three dimensions: color, orientation, and shape. Their visual scenes manipulated similarity (i.e., the number of shared features between target and distractors) and grouping (i.e., the number of groups of identical distractors). The results indeed revealed effects of grouping: fewer groups led to more efficient search. For similarity, which is one of the classical laws of grouping, the authors found that search times increased as the distractors shared more features with the target. With these findings, Nordfang and Wolfe (2014) show that visual scene perception is indeed guided by grouping strategies, which can hinder or facilitate visual search, depending on the form of grouping that is dominant in a particular visual scene.

The current paper does not investigate perceptual grouping from the receiver's side, which could be the listener in a referential communication task, but from the production side: how does it guide speakers' perception of a scene, and affect the references that they produce? In Experiment 1, one classical principle (*similarity*), and one more recent principle of grouping is manipulated (*common region of space*). Similarity holds that the most similar elements are grouped together (Wertheimer, 1923). Following this principle, the two bowls in Figure 1 could form a group, because they look the same and have the same type. However, the yellow plate has a different type (and shape), and may thus fall in a different group. In the current paper, this is referred to as *type similarity*. The second principle of grouping that is manipulated in Experiment 1, common region of space, was introduced by Palmer (1992), as an addition to the more classical laws of grouping by Wertheimer (1923). In Palmer's (1992) definition, the region of space principle holds that entities that fall within an enclosing contour are usually perceived as a group. For example, in an expression like “*the silverware on the counter,”* the counter is the enclosing contour, and the pieces of silverware are the objects that are grouped together. Similarly, in Figure 1, two of those enclosing contours can be distinguished, namely the sideboard and the table surface.

The question is to what extent type similarity and region of space guide speakers in restricting the set of relevant distractors in a visual scene. This study provides systematic manipulations of these two grouping principles to answer this question. For both principles, it is argued that objects that are in the same perceptual group as the target are more likely to be in the speakers' focus of attention (in the sense of Beun and Cremers, 1998), and therefore more likely to be considered a relevant distractor. Along similar lines, the opposite is expected for distractors that fall in a different group than the target. The result would be that speakers would not consider the yellow plate a relevant distractor in Figure 1, since it has a different type than the target, but also because it is in a different region of space (i.e., sideboard rather than table surface). Similarly, it can be argued that the types and surfaces of the two bowls are shared, which could make the large bowl a relevant distractor.

For Experiment 1, it is hypothesized that the perception of groups influences the content of the referring expressions that speakers produce. Echoing previous work on guided search (e.g., Itti and Koch, 2001; Wolfe and Horowitz, 2004; Wolfe, 2010), we know that speakers are (far) more likely to redundantly include color attributes in their object descriptions in polychrome rather than monochrome displays (Koolen et al., 2013; Rubio-Fernández, 2016). Thus, if type similarity and region of space cause speakers to limit the set of relevant distractors for the target in Figure 1 to the two bowls, it is unlikely that color is added. After all, only two bowls are left in that case, both green, so monochrome. A description without color (“*the small bowl”*) is then likely to be produced. However, if the plate had been a bowl, or if it had been placed on the table rather than the sideboard, the distractor set may become bigger, and thus polychrome. An overspecified description with color (“*the small green bowl”*) would then most likely be uttered. For type similarity, Koolen et al. (2016) indeed found such an effect of grouping on overspecification with color, so the current manipulation of type similarity was included as a replication.

## Presentation Mode: 2D vs. 3D Scenes

The second factor that is expected to affect people's perception of distractor distance relates to the *mode* in which the visual scenes are presented to them: 2D or 3D. Comparing these presentation modes is particularly relevant for research on visually-grounded language production and referential overspecification. The majority of previous experiments in this direction presented participants with artificial visual scenes, consisting of drawings of objects that are configured in grids (e.g., Pechmann, 1989; Koolen et al., 2013; Rubio-Fernández, 2016; Van Gompel et al., 2019; among many others). Some previous work used realistic photographs as stimuli (e.g., Coco and Keller, 2012; Koolen et al., 2016), but these were 2D representations of 3D scenes.

A closer look at previous work in the field of visual search gives reason to believe that reference production studies would indeed benefit from using more naturalistic scenes, mainly because the (usually) higher visual and semantic complexity of naturalistic scenes affects the visual search process. More specifically, coherent naturalistic scenes may trigger expectations about what the “gist” of the scene could be. This initial understanding of a scene can be a strong guide when searching for specific objects in a scene (e.g., Torralba et al., 2006; Wolfe and Horowitz, 2017), and reduce the impact of low-level visual information (Wolfe et al., 2011a,b). A concrete example of how more natural scenes may affect visual search comes from Neider and Zelinsky (2008), who found that adding more objects to quasi-realistic scenes actually leads to shorter visual search times, and different fixation patterns, for example due to perceptual grouping processes. Therefore, in an attempt to address these findings in reference production, to further enhance ecological validity, and because distances between objects may be perceived differently in 3D rather than 2D (as argued below), the current study tests the effect of presentation mode on reference production systematically.

In perceiving depth and distance information, people normally rely on a combination of *monocular* and *binocular* depth cues. Examples of monocular depth cues are—among others—relative size of objects, shading, occlusion, and perspective (McIntire et al., 2014). Monocular cues can be easily seen with one eye, even in 2D visual scenes such as paintings and photographs. However, in cases where viewers are presented with flat, 2D imagery where monocular cues are degraded or ambiguous, depth perception, and estimations of distance may suffer (McIntire et al., 2014). Therefore, ideally, viewers could not only rely on monocular cues, but also on binocular cues, which can only be perceived with two eyes (Loomis, 2001). After all, the use of two eyes allows viewers to view the world from two different angles (one for each eye), and delivers them with two perspectives on the situation. These two “half-images” are then combined into one coherent “stereo pair”; this process is called *stereopsis* (McIntire et al., 2014). For perceiving depth, and for extracting the distance to (and between) objects, the difference between the two half-images is crucial. For example, for perceived objects that are far away, the difference between the half-images is relatively small, while it is bigger for closer objects.

The phenomenon of stereopsis is also applied in artificial 3D presentation techniques, whose technologies allow them to present viewers with two half-images, combined with monocular depth cues, all in a single display system (McIntire et al., 2014). However, when looking at previous research on visually-grounded reference production throughout the years, such techniques are hardly used. Instead, most (if not all) scholars tend to present participants with flat, 2D representations 3D visual environments, sometimes even in the form of abstract drawings (e.g., Pechmann, 1989; Koolen et al., 2013; among many others). Although there is some previous work on visual perception showing that people normally have no difficulty in understanding the three-dimensional nature of 2D images (Saxena et al., 2008), the question is to what extent the lack of binocular depth cues may affect people's estimations of distance between objects for those images. For one thing, it is possible that 2D visual scenes lead to poorer estimations of distance between a target and its surrounding distractors, which may in turn have repercussions for the distractor set that speakers rely on in a reference production task.

In the early scientific literature on the perception of 2D vs. 3D displays, it has been shown that binocular depth perception is more accurate than monocular depth perception, for infants (Granrud et al., 1984), but also for adults (Jones and Lee, 1981). For example, Jones and Lee (1981) found a superior effect of binocular vision in several tasks that require precise depth perception, such as reaching for an object. These findings are referred to in a recent review article by McIntire et al. (2014), who discuss whether 3D presentation mode should always be preferred over 2D. In their review, McIntire et al. distinguish between different kinds of tasks, including judgments of position and distance, visual search, and spatial understanding. Their general impression is that 3D representation can be beneficial, but not for all tasks. However, for tasks related to the experimental task in the current study, which requires participants to decide on the relevance of certain distractors in a reference production task, 3D displays seem to be preferred. For example, of the 28 reviewed studies that measured position judgments or distances of displayed objects, 16 studies showed a clear benefit of 3D over 2D. The pattern was even more convincing for visual search tasks, where it was found that the detection of targets in cluttered scenes is easier in 3D than in 2D displays, at least for static targets (McKee et al., 1997). Finally, the most convincing performance benefit of 3D displays was found for experiments where participants had to move (or otherwise manipulate) virtual or real objects (McIntire et al., 2014).

In their general discussion, McIntire et al. (2014) conclude that 3D displays are most beneficial for depth-related tasks that are performed in close spatial proximity to the viewer. For the current paper, I would like to argue that a reference production task for a target in, say, Figure 1 is such as a task. After all, Figure 1 depicts a living room in which the objects are quite close to the viewer. For closer objects, Cutting and Vishton (1995) found that they lead to a relatively larger benefit of binocular cues over monocular cues when perceiving depth and distance, because the difference between the two half-images is bigger. Therefore, it is expected that speakers are better able to accurately perceive the distance between the target and its distractors in a 3D representation of Figure 1, rather than a 2D representation. This may also affect speakers in determining the set of relevant distractors for a visual scene. For example, in Figure 1, the plate on the sideboard may be considered a relevant distractor in 2D, but not in 3D, because poorer estimations of distance may cause speakers to rely on a bigger distractor set in the former case. Thus, in 2D, speakers may consider the plate, just to be sure, since it is more difficult for them to decide if it is a relevant distractor or not. The result could then be an overspecified expression, because color variation (yellow plate, green bowl) causes speakers to use color (Koolen et al., 2013; Rubio-Fernández, 2016).

## Experiment 1

The first experiment was a reference production experiment, in which participants were presented with scenes like the one displayed in Figure 1, and had to produce uniquely identifying descriptions of the target referents. The scenes were set up in such a way that color was never needed to identify the target. This made it possible to take the proportional use of redundant color attributes as the dependent variable. There were two presentation modes to present the stimuli to the participants (2D and 3D), and two manipulations of perceptual grouping: one by having different distractor types (same or different than the target), and one by systematically placing one distractor either in the same region as the target, or in a different one. As explained later on, the manipulated distractor always had a different color than the target.

### Method

#### Participants

Forty-eight undergraduate students (33 female; mean age: 21.6 years) from Tilburg University took part in the experiment for course credit. All were native speakers of Dutch, the language of the experiment. All participants signed a written informed consent form, which was approved by the ethics committee of the Tilburg Center for Cognition and Communication (Tilburg University). A positive evaluation of the experiment and the study protocol were part of this approval.

#### Materials

The stimulus materials were near-photorealistic visual scenes, modeled, and rendered in Maxon's Cinema 4D (a 3D modeling software package). There were 72 trials in total, all following the same basic set-up: participants saw a picture of a living room that contained a dinner table and a sideboard, plus some clutter objects for more realism. The table and the sideboard formed two surfaces on which objects were positioned: one target object and two distractor objects were present in every scene. The target object always occurred at one side of the table, in the middle of the scene. The first distractor object was always placed close to the target, either on the left or the right side. This distractor had the same type and color as the target, meaning that it could only be ruled out by means of its size. Crucially, there was also a second distractor object in every scene. This distractor always had a different color than the target, to induce overspecification with color. The second distractor was used to apply two manipulations of perceptual grouping: region of space, and type similarity. Therefore, this distractor is hence referred to as the “manipulated distractor.” The exact manipulations, as well as a third factor (presentation mode) are explained in more detail below.

**Figure 2:**
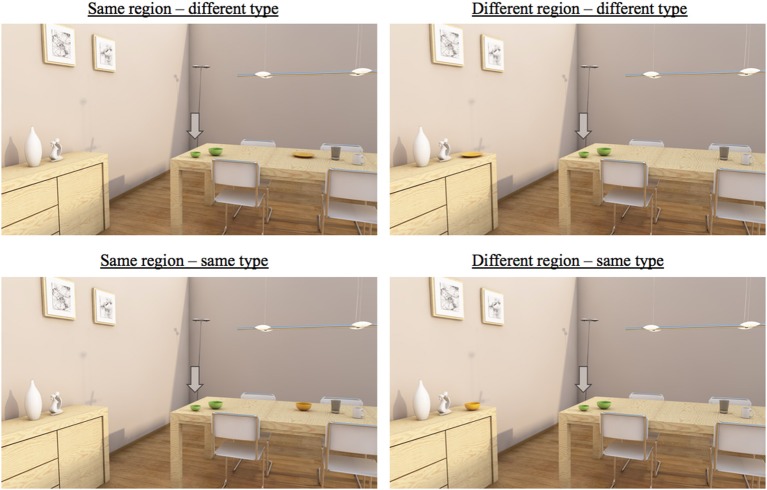
Examples of critical trials (in 2D). The left scenes are trials in the same group condition, while the right scenes are trials in the different group condition. The upper scenes are trials in the different type condition, while the lower ones are trials in the same type condition. Note that the trials were presented to the participants on a big television screen, and that manipulations of color may not be visible in some print versions of this paper.

Firstly, there was a manipulation of *region of space*. This factor was manipulated as follows: in half of the trials, the manipulated distractor and the target object were in the *same* region (meaning that they were both positioned on the table), while they were in a *different* region in the other half of the trials (with the target placed on the table, and the distractor on the sideboard). Example scenes for these two conditions can be found in Figure 2. The left scenes represent the *same* group condition: in these scenes, all objects are on the table. The right scenes represent the *different* group condition: the target object (the small bowl) is again on the table, while the manipulated distractor (i.e., the plate in the upper picture, and the yellow bowl in the lower picture) is placed on the sideboard. Crucially, the distance between the target and the manipulated distractor was always the same in the two conditions.

The second manipulation of grouping was *type similarity*: the *type* of the manipulated distractor in the scene could either be different or the same as the target's type. For example, in Figure 2, the manipulated distractor (the plate) has a different type than the target (the bowl) in the upper two trials, while all relevant objects have the same type in the lower trials. Note also that mentioning a target's type and size was sufficient to distinguish the target in all four scenes, implying that the use of color would always result in overspecification. This applied to all scenes used in the experiment.

The experiment consisted of 72 trials, 16 of which were critical trials. As said, in the critical trials, all scenes had the same basic set-up, but four different sets of objects were used as target and distractor objects. In Figure 2, trials for one of these sets are depicted, with bowls and a plate. Regarding the other sets, it was made sure that they all consisted of food-related objects (such as mugs and cutting boards) that can reasonably be found on a sideboard or a dinner table in a living room. Since the target was always accompanied by a distractor object of the same type, the size of the target was varied: in two sets of objects, the target was the bigger object of the two; in the other two sets, the smaller object was the target. This way, it was aimed to avoid the occurrence of repetition effects: speakers could not stick to referring strategy, for example by always mentioning that the target was “small.”

The scenes for the 4 sets of objects were manipulated in a 2 (*region of space*) × 2 (*type similarity*) design, which resulted in four within conditions as described above: one scene in which the manipulated distractor object shared a group with the target, but not its type; one in which that distractor shared its group and its type with the target object; one in which the manipulated distractor neither shared a group, nor its type with the target; and one in which the distractor did not share its group with the target, but did share its type.

On top of region of space and type similarity, which were both manipulated within participants, there was also one between factor: *presentation mode* (2D/3D). Participants were randomly assigned to either the 2D or the 3D condition. In the 2D condition, the trials were presented to the participants as flat 2D images (i.e., regular photos). As explained in the introduction section of this paper, for 2D images, a viewer depends solely on monocular cues to perceive depth information, and distance between objects in particular. In the 3D condition, the trials were presented as 3D images, where speakers could rely on both monocular and binocular depth cues to perceive depth information. The visual scenes in the 2D condition were rendered in the same way as those in the 3D condition, but the image for the left eye was 100% identical to that for the right eye, eliminating depth differences. This means that the 2D and 3D scenes were neither different in the objects that were visible, nor in the positioning of these objects in the scenes. The size of the stimuli was the same in the two conditions.

**Figure A1:**
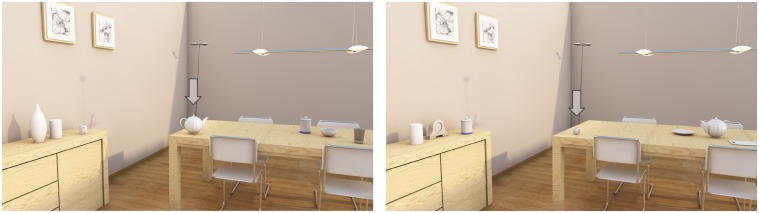
Two example fillers that were used in Experiment 1.

The experiment had 56 fillers, all following the basic setup of the critical trials, with the table and the sideboard, with the target object positioned in the middle of the scene. Again, different kinds of objects were visible, close or distant to the target that had to be described by the participants. However, crucially, color differences between target and distractors were avoided, so that the speakers were not primed in using color attributes when describing the filler targets. For this purpose, the fillers trials mainly contained white or transparent objects (see Figure A1 for two example fillers). In order to avoid learning effects, the number of fillers was relatively high.

#### Procedure

The experiment took place in an office room at Tilburg University, and participants took part one at a time. The running time for one experiment was ~15 min. After participants had entered the room, they were randomly assigned to the 2D or 3D condition (24 participants each). They were then asked to sit down and read an instruction manual. The manual explained to the participants that they would be presented with scenes in which one of the objects was marked with an arrow. This target had to be described in such a way that a listener could distinguish it from the other objects that were present in the scene. Once participants had read the instructions, they were given the opportunity to ask questions.

The participants (all acting as speakers in the experiment) were seated in front of a large 3D television, while wearing 3D glasses. This was done regardless of the condition they were assigned to, to eliminate differences in the procedure. In the 2D condition, the television displayed flat 2D images of the stimuli. In the 3D condition, the TV used “active” 3D technology to display the trials: it synchronized with the 3D glasses by means of infrared signals, and used electronic shutters to separate images through the participant's right and left eye. The three-dimensional input was configured as side-by side: both eyes would view an image with a source resolution of 960 × 1,080 pixels, presented on an LCD panel with a resolution of 1,920 × 1,080 pixels. The scenes were presented as still images at 120 Hz, resulting in 60 Hz per eye. In both conditions, participants were shown a short introduction movie (a fragment from the “Shreck” or “Ice Age” movies), so that they could get accustomed to the TV and the glasses.

There were two versions of the experiment in terms of trial order: there was one block of trials in a fixed order that was determined by a randomizer. This block was presented to half of the participants. A second block of trials took the reverse order, and was presented to the other half of the participants. The trials were set as slides, and presented using Keynote. No transitions or black screens were used; when a trial was completed, the transition to the next trial was instant. The participants could take as much time as needed to provide a description for every target object, and their descriptions were recorded with a voice recorder. The listener, who was a confederate of the experimenter, sat behind a laptop (out of the speaker's sight), and clicked objects he thought the speaker was referring to. Each time the listener had done this, the next trial appeared. The confederate was always the same person (male, 26 years old), and he was instructed not to click on the target before he was sure that the speaker had finished her description. The speaker's instructions indicated that the positioning of the objects was different for the listener. This eliminated the use of location information as an identifying attribute, avoiding descriptions such as “*The bowl at the right side of the table.”* The confederate listener never asked clarification questions, so that the speakers always produced initial descriptions.

#### Design and Statistical Analysis

The experiment had a 2 × 2 × 2 design with two within-participants factors: *region of space* (levels: same region, different region) and *type similarity* (levels: same type, different type), and one between-participants factor: *presentation mode* (levels: 2D, 3D). The dependent variable was the proportional use of redundant color attributes. As described above, using color was never required to distinguish the target from its distractors: mentioning size always ruled out the first relevant distractor, while adding the target's type eliminated the other relevant distractor. Thus, if speakers used color anyway, this inevitably resulted in overspecification.

The statistical procedure consisted of Repeated Measures ANOVAs: one on the participant means (*F*1) and one on the item means (*F*2). To generalize over participants and items simultaneously, also *minF*′ was calculated; effects were only regarded as reliable if *F*1, *F*2, and *minF*′ were all significant. To compensate for departures from normality, a standard arcsin transformation was applied to the proportions before the ANOVAs were run. For the sake of readability, the untransformed proportions and analyses are reported in the results section, also because they revealed the exact same patterns of results. Similarly, again for readability, interaction effects are only reported on when significant. Table A1 provides the results for the (non-significant) interaction effects that are not reported in the main text.

### Results

A total of 768 descriptions were produced in the experiment for the critical trials. Speakers mentioned a redundant color attribute in 66.0% of the cases. The order in which the trials were presented to the participants (regular vs. reversed) had no effect on the use of color (*p* = 0.33), and is therefore not further analyzed below. Table 1 provides an overview of the means and standard errors for the redundant use of color, as a function of the main effects of the independent variables.

**Table 1:** The means and standard errors for the redundant use of color in Experiment 1, as a function of all the main effects analyzed.

	**Presentation mode**		**Region of space**		**Type similarity**	
	**2D**	**3D**	**Same**	**Different**	**Same**	**Different**
Redundancy	0.75 (0.01)	0.57 (0.02)	0.69 (0.02)	0.63 (0.02)	0.69 (0.02)	0.63 (0.02)

#### Results for Presentation Mode

It was first examined if the way in which the trials were presented to the participants (i.e., in 2D or in 3D) had an effect on the redundant use of color. The results show that the presentation mode to some extent affected the use of the redundant attribute color, but this effect was only significant by items [*F*1_(1, 46)_ = 2.73, n.s.; *F*2_(1, 12)_ = 39.71, *p* < 0.001, ${\eta_{\text{p}}}^{2}$ = 0.77; *minF*′_(1, 52)_ = 2.55, n.s.]. This means that the speakers in the 2D condition included color more often than speakers in the 3D condition, but that there was no reliable effect of presentation mode. See Table 1 for the means.

#### Results for Common Region of Space

The second factor that was expected to have an effect on the redundant use of color was a manipulation of perceptual grouping: region of space. The results indeed showed an effect of region of space on the redundant use of color [*F*1_(1, 46)_ = 7.81, *p* = 0.008, ${\eta_{\text{p}}}^{2}$ = 0.15; *F*2_(1, 12)_ = 9.02, *p* = 0.01, ${\eta_{\text{p}}}^{2}$ = 0.43; *minF*′_(1, 41)_ = 4.18, *p* < 0.05]. As predicted, proportions of color use were higher in the *same* group condition than in the *different* group condition (see also Table 1). Overall, this means that speakers were more likely to include color in scenes where all objects were positioned on the table, as compared to the scenes in which the manipulated distractor was placed on another surface (i.e., the sideboard).

**Figure 3:**
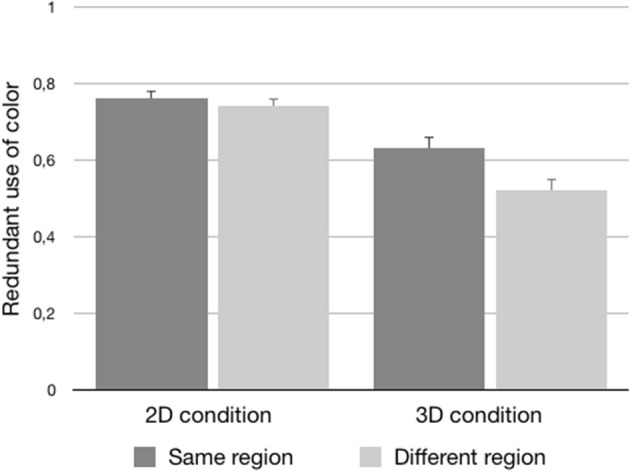
The proportional use of color (plus standard errors) for the 2D and 3D conditions as a function of the same group and different group stimuli.

Further inspection of the data suggests that this effect of region of space was stronger for 3D stimuli rather than 2D stimuli. As visualized in Figure 3, in the case of the 2D stimuli, there was hardly a numerical difference between the *same* group condition (*M* = 0.76, *SE* = 0.02) and the *different* group condition (*M* = 0.74, *SE* = 0.02), while this difference was bigger for the 3D stimuli (*same* group condition: *M* = 0.63, *SE* = 0.03; *different* group condition: *M* = 0.52, *SE* = 0.03). However, this interaction between perceptual grouping and presentation mode only reached significance by participants [*F*1_(1, 46)_ = 4.61, *p* = 0.04, ${\eta_{\text{p}}}^{2}$ = 0.09; *F*2_(1, 12)_ = 2.97, n.s.; *minF*′_(1, 29)_ = 1.80, n.s.]. Therefore, this interaction was not statistically reliable.

#### Results for Type Similarity

Finally, a second manipulation of grouping was tested: type similarity. It was found that the type of the manipulated distractor indeed had an effect on the redundant use of color [*F*1_(1, 46)_ = 6.88, *p* = 0.01, ${\eta_{\text{p}}}^{2}$ = 0.13; *F*2_(1, 12)_ = 9.09, *p* = 0.01, ${\eta_{\text{p}}}^{2}$ = 0.43; *minF*′_(1, 44)_ = 3.91, *p* = 0.05]. The means show that speakers more often used color when the distractor's type was the same rather than different (see Table 1 for the means). Type similarity did not interact with the other factors that were manipulated.

### Interim Discussion

In the first experiment, I investigated how the perceived distance between objects in a scene affects speakers' production of definite object descriptions, and, in particular, to what extent it causes them to include redundant color attributes in such descriptions. Three factors were tested: two manipulations of perceptual grouping (type similarity and region of space, and a manipulation of presentation mode; 2D vs. 3D).

As predicted, there were effects of perceptual grouping on the redundant use of color. Firstly, there was a main effect of type similarity, similar to the one reported on earlier by Koolen et al. (2016): speakers included color more often when a target and its distractor had the same type (e.g., two bowls) rather than different types (e.g., a bowl and a plate). These findings suggest that a distractor is more likely to be considered a relevant distractor if it shares its type with the target, as compared to when this is not the case. Since the target and the distractor had a different color in the current stimuli, the assumed bigger distractor set led to color variation, and thus to the selection of more redundant color attributes (Koolen et al., 2013; Rubio-Fernández, 2016).

Also the manipulation of grouping, common region of space (Palmer, 1992), resulted in a significant effect on overspecification with color. It was expected that objects that are in the same region of space as the target are more likely to be considered a relevant distractor than objects in a different region. To test this assumption, the stimuli systematically placed one distractor (the one with the different color) either in the same region as the target (i.e., on the table), or in a different one (i.e., the sideboard). The distance between the objects was the same. As hypothesized, participants used color more often in the same region condition than in the different region condition, which suggests that in the former case, the differently colored object was more likely to be in a speakers' focus of attention (in the sense of Beun and Cremers, 1998). These findings suggest that speakers indeed perceive objects around them in groups, and that this tendency guides them in determining the distractor set in a reference production task.

Although the interaction effect between region of space and presentation mode was not fully reliable, it should be noted that the above effect of region of space seemed to be stronger in 3D visual scenes; in 2D, the numerical difference between the proportional use of color in the same and different region conditions was really. This pattern suggests that the combination of monocular and binocular depth cues in 3D (Loomis, 2001) may enhance effects of grouping. This is somewhat surprising, since previous literature has revealed that the presence of binocular cues leads to better performance on many tasks (McIntire et al., 2014), and also to better estimations of depth and distance, especially for close objects such as the ones in the visual scenes manipulated here (Cutting and Vishton, 1995). For that reason, one would expect that the binocular cues in 3D lead to a more restricted distractor set, since better estimations of distance may also lead to more accurate perception and formation of groups. Thus, in 3D, speakers may be better able to “see” that the distance between the target and the manipulated distractor in the two region of space conditions was in fact the same, which should make redundant color use comparable for the two conditions. However, this was clearly not the case, as the interaction was not fully reliable, and the means showed the opposite pattern. This pattern, and the lack of a main effect of presentation mode, are discussed in more detail in the section General Discussion.

Experiment 1 revealed interesting effects on the interplay between visual scene perception and reference production. However, there is at least one relevant limitation: like many previous studies on visually-grounded reference production (e.g., Clarke et al., 2013a; Koolen et al., 2013; Rubio-Fernández, 2016, among many others), it has applied an indirect measure of visual attention, in this case the occurrence of overspecification with color. This approach has its limitations when studying how the distractors in a scene shape the content of referring expressions. For example, although the results of Experiment 1 show that region of space and type similarity affect overspecification, there is no direct evidence that these results are due to the way in which speakers ignore certain distractors that are in a different region than the target referent, or that have a different type. Therefore, Experiment 2 collects eye movements as a direct, online measure of visual attention, and combines these data with a more traditional, offline analysis of referential overspecification. The focus in the experiment is on perceptual grouping: it reconsiders the effects of region of space and type similarity, and adds a third grouping principle: *color similarity*. Experiment 2 does not reconsider the effect of presentation mode, due to the technical challenges of measuring eye movements in a 3D paradigm. Please note that this is also the reason why an offline measure of visual perception was applied in the first experiment.

While eye-tracking methodologies are commonly used to investigate language comprehension (e.g., Tanenhaus et al., 1995), they are still comparatively rare in language production research, initially because speech movements can disrupt eye movement data (Pechmann, 1989; Griffin and Davison, 2011). After some early studies that explored the effect of object fixations on order of mention (e.g., Meyer et al., 1998; Griffin and Bock, 2000), also for event descriptions (Gleitman et al., 2007), some researchers recently started to apply eye-tracking to test the effects of perceptual and conceptual scene properties on rather open-ended scene descriptions (Coco and Keller, 2012, 2015), object naming (Clarke et al., 2013b), and image captions (Van Miltenburg et al., 2018). Also visually-grounded reference production has been explored in eye tracking experiments, with child participants (Rabagliati and Robertson, 2017; Davies and Kreysa, 2018) and adult participants (Brown-Schmidt and Tanenhaus, 2006; Vanlangendonck et al., 2016; Davies and Kreysa, 2017; Elsner et al., 2018). However, none of this work has tested systematically how various types of perceptual grouping shapes attribute selection during reference production.

## Experiment 2

In the second experiment, participants had the same task as in Experiment 1: they produced uniquely identifying descriptions of target objects that were presented to them in 2D visual scenes. Also the basic set-up of the scenes was the same: there was a living room with the same two surfaces, with three crucial objects, including the target, and a distractor with which the factors under study were manipulated. Both the participants' speech as well as their eye movements were recorded during the reference production task. The speech data were annotated for the occurrence of overspecification; i.e., if descriptions contained a redundant color attribute. New in this experiment are the eye tracking data. Here, I analyzed the number of fixations on the manipulated distractor, and the total gaze duration for that object.

As mentioned above, the experiment applies a manipulation of common region of space, as well as two manipulations of similarity: color similarity and type similarity. Adding color similarity as a factor in the design is a relevant thing to do, since we know that speakers overspecify more often in polychrome rather than monochrome displays (Koolen et al., 2013; Rubio-Fernández, 2016). The current study further explores this finding in an eye tracking paradigm. Furthermore, for overspecification with color, color similarity has been found to interact with type similarity: the proportion of overspecification is highest when there is at least one distractor object that shares its type with the target, but not its color (Koolen et al., 2016). Again, it is relevant to test how this interaction is reflected in speakers' eye movement data.

For region of space, it is hypothesized that if a distractor is in the same region of space as the target, it will be viewed more often and longer than if the region of space is different, and that this will eventually lead to more overspecification with color. The same goes for type similarity, with more views, longer viewing time and more overspecification for a distractor of the same rather than a different type than the target. Thirdly, for color similarity, it is hypothesized that a distractor most likely attracts attention if it has a different color than the target, resulting in more views, longer viewing times, and again more overspecification than for a distractor that shares its color with the target. Finally, type similarity and color similarity may interact: distractors with the same type and a different color than the target are expected to lead to the highest proportions of overspecification, most fixations, and longest viewing times.

### Method

#### Participants

Thirty-one participants (26 female, mean age: 21.6) took part in the experiment. These participants had not taken part in Experiment 1, were gathered randomly at the campus of Tilburg University, and received a piece of candy as a reward. All participants were native speakers of Dutch, which was again the language of the experiment. Again, all participants signed a written informed consent form, which was approved by the ethics committee of the Tilburg Center for Cognition and Communication (Tilburg University). Like in the first experiment, a positive evaluation of the experiment and the study protocol were part of this approval.

#### Materials

The stimulus materials were the same as the 2D visual scenes used in Experiment 1, except that one extra manipulation was added: color similarity (see below). In general, the scenes depicted the same living room with a dinner table, a sideboard, and some clutter objects. The table and the sideboard again formed the two surfaces (i.e., regions of space) that were important for the manipulations, since these were again the spaces where the target and its two distractors were positioned. Like in Experiment 1, the target object was always placed on the table, in the middle of the scene, together with a distractor right next to it (either left or right). This first distractor object had the same type and color as the target object, but a different size. So, again, mentioning size was always sufficient for a distinguishing description, and using color thus resulted in an overspecified description.

**Figure 4:**
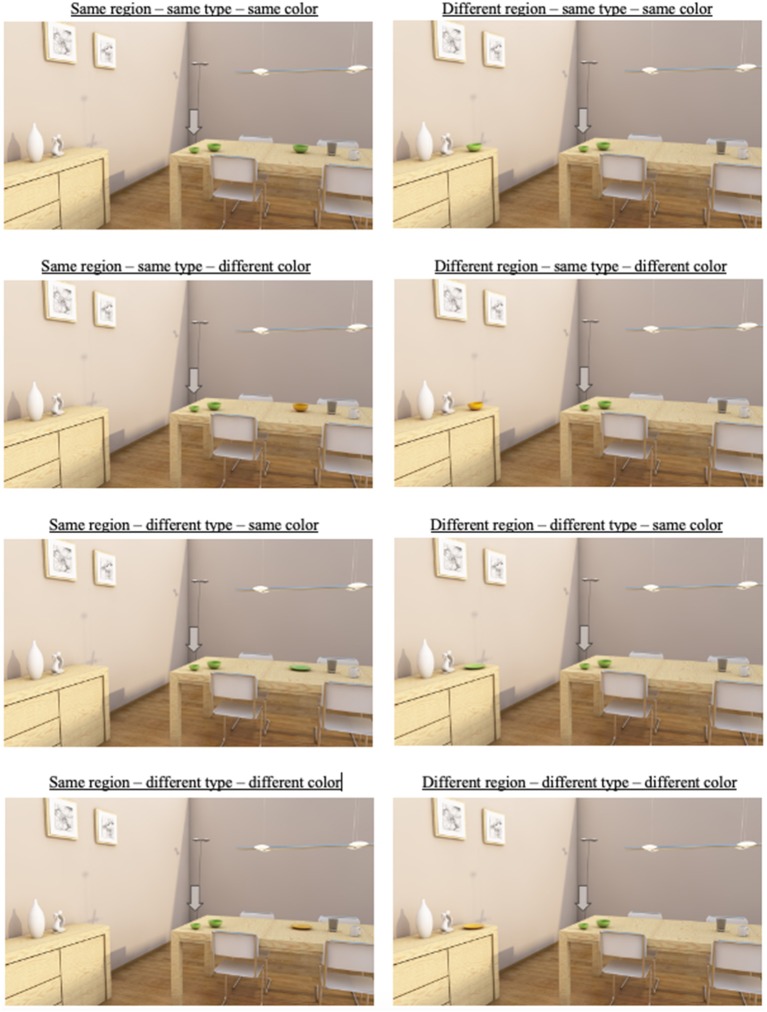
Examples of critical trials in the experiment. The distractor shares its region of space with the target (i.e., the table) in the left scenes, and is in a different region (i.e., the sideboard) in the right scenes. The distractor has the same type as the target in the upper four pictures, and a different type in the lower four pictures. The distractor has the same color as the target in the first, second, fifth, and sixth picture, and a different color in the third, fourth, seventh, and eighth picture. Note that manipulations of color may not be visible in some print versions of this paper.

The manipulations of grouping were again realized by manipulating with one specific distractor object, hence referred to as the “manipulated distractor.” The manipulations of *region of space* and *type similarity* were replications of Experiment 1: the manipulated distractor occurred in the same or a different region, and had the same or a different type. The manipulation of *color similarity* was new in the design: the color of the manipulated distractor could be the same as or different than the color of the target. In Figure 4, example scenes of all the different conditions can be found, for one specific target: a bowl. There were similar manipulations for three other types of targets: a plate, a mug and a cutting board. Participants were thus presented with 32 (four types × eight conditions) critical trails. This means that there were four cells per condition for each participants.

Three measures were taken to avoid participants from using the same strategy for all critical trials. Firstly, there were 32 filler trials, which were taken from Experiment 1, with objects in various configurations. Secondly, two versions of the experiment were created. For both versions, half of the visual scenes for the critical trials were mirrored. In version 1, this was done for the scenes in which the manipulated distractor was in the same region of space as the target object, while in version 2, all scenes in the different region of space condition were mirrored. In practice, this means that the sideboard—which represented the “different” region of space area—was either positioned left or right from the target, depending on version and condition. By mirroring half of the critical trails, it was avoided that speakers could strategically start looking for the manipulated distractor object in the same area of the image; this object could now occur in two different areas (i.e., left or right from the target).

#### Procedure

This second experiment took place in a soundproof booth, located in the research laboratory at Tilburg University. The eye-tracking measurements were made with a SMI RED250 device, operated by the IviewX and the Experiment Center software-packages. The eye-tracker had a sampling rate of 250 Hz. The microphone of a webcam was used to record the object descriptions of the participants; the camera was taped off for privacy reasons. The stimulus materials were displayed on a 22 inch P2210 Dell monitor, with the resolution set to 1,680 × 1,050 pixels, with 90.05 pixels per square inch.

After entering the laboratory, participants signed a consent form, and read a first basic instruction stating that they were going to act as the speaker in a language production experiment. Participants were then seated in the soundproof booth, in front of the eye tracker, and their eyes were calibrated using a 9-point validation method. When the calibration was completed successfully, participants were invited to read a second instruction, which was essentially the same as the one provided in Experiment 1. It stated that participants were going to produce spoken descriptions of target objects in visual scenes in such a way that these objects could be distinguished from the remaining objects in the scene. Again, it was emphasized that using location information in the descriptions (e.g., “the bowl on the left”) was not allowed. After this second instruction, participants completed two practice trials, and had the possibility to ask questions. Once the procedure was clear, the experimenter left the booth, and the experiment started.

All participants were shown a total of 64 stimuli (32 critical trails and 32 fillers) in a random order. The visual scenes were depicted in the middle of the screen, filling 70% of the available space; the remaining 30% consisted of a gray border surrounding the scenes. Before every trial, a screen with an “X” appeared somewhere in the 30% contour area. When this X had been fixated for 1 s, the next visual scene appeared automatically. When fixating the X did not work, participants could make the next scene appear manually by pressing the spacebar. The position of the X was different for all trials: they appeared in a random position in the gray border, to make sure that participants did not develop a viewing strategy. There were 1.6 times more X triggers on the top and bottom row than on the left and right side, in proportion to the 1,680 × 1,050 screen resolution. Since the location of the fixation cross was picked randomly for every trial and every participant, specific locations of the fixation cross were not linked to particular scenes or conditions. Once all 64 trials had been completed, participants were instructed to leave the booth. It took 30 min on average to complete the experiment.

#### Research Design

The experiment had a 2 × 2 × 2 design with three within-participants factors: *region of space* (same, different), *type similarity* (same, different), and *color similarity* (same, different). Three dependent variables were measured: the overspecification with color; the gaze duration upon the manipulated distractor in milliseconds per trial per participant; and the number of times that the manipulated distractor was fixated per trial per participant.

#### Data Coding and Preparation for Analysis

**Figure 5:**
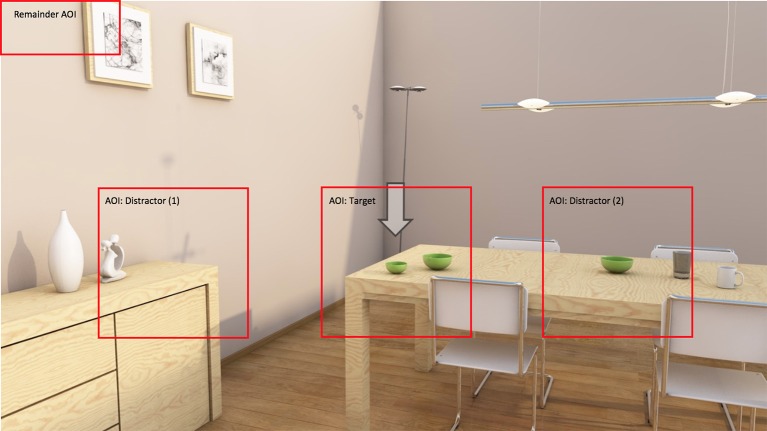
An overview of the areas of interest that fixations were assigned to. Note that the left and right AOI were central to the analyses, depending on condition.

All recorded object descriptions were transcribed and coded for the presence of color (0 or 1). For the eye-tracking data, all fixations were assigned to one of the areas of interest (AOIs) that were defined. There was one AOI for the target, one for the sideboard, one for the central part of the table, and one remainder area (see Figure 5). The AOIs for the sideboard and the central part of the table represented the areas where the manipulated distractor could be placed, depending on condition. These two AOIs were central to the analyses. As can be seen in Figure 5, these AOIs were relatively big, in line with the recommendations by Orquin et al. (2015). This was possible because the distance between the objects in the scenes was large, avoiding the risk of falsely positive assignments of fixations.

For trials such as the one in Figure 5, which has the manipulated distractor placed on the table, fixations within the right AOI were used in the analyses. Similarly, fixations within the left AOI where used for trials with the distractor on the sideboard. Fixations on the target object were assigned to the target AOI, but not analyzed. The remainder area was used for fixations that were not on the target or any of the distractor objects. For one participant, there turned out to be calibration problems with the eye-tracker, so the data recorded for this person was excluded from further analysis.

The coding process resulted in a separate path file for every participant. These path files were converted into a single file, and loaded into SPSS for statistical analysis. Although there was supposed to be data for 960 target descriptions (30 speakers × 32 trials), the data for 24 trials could not be analyzed because either the description or the eye movements were not recorded correctly. The final analysis thus contained data for 936 trials (i.e., 97.5% of total data). For the analyses of overspecification, data for all 936 trials were used; for gaze duration and the number of fixations, subsets of the data were created. For these two variables, only the cases where speakers fixated—and thus saw—the manipulated distractor were analyzed. This was the case in 680 out of 936 cases (i.e., 72.6% of total data). In the section General Discussion, this approach is reflected upon.

For gaze duration, I then calculated for every trial the total amount of time that the participant looked at the manipulated distractor, and standardized this score by calculating the *z*-score per trial per speaker. Only scores in the range of −3 ≤ *z* ≤ 3 were included in the analysis, which means that scores for 13 cases were filtered out. For the number of fixations, a similar subset of the data was created, but this time I calculated the number of times that speakers looked at the manipulated distractor object for every trial. Again, the *z*-score was calculated, which led to the exclusion of 12 trials that were not part of the final analysis for this dependent variable. For both variables, two types of analyses are provided: one on the absolute scores (i.e., exact fixation duration and exact number of fixations), and one on the relative scores (i.e., gaze duration on the distractor divided by total gaze duration; number of fixations on the distractor divided by the total number of fixations).

### Results

To test for significance, again three repeated measures ANOVA tests were performed: one for every dependent variable. For the ANOVA testing the effects of redundant color use, data for all 30 participants was used (936 cells; participant range: 24–32). This was not possible for the other two ANOVAs, since subsets of the data were used there, leading to a substantial amount of empty cells (see section Data Coding and Preparation for Analysis). There were six participants with missing data in one or more conditions after aggregating the scores for every condition separately. These participants were excluded from the analyses for the two eye tracking variables, leaving data for 24 participants there. For both the number of fixations and gaze duration, the total number of cells analyzed was 611, with a participant range of 16–32 (the maximum was 32). Like in Experiment 1, interactions are only reported on when significant; the results for the (non-significant) interaction effects that are not reported in the main text are in Table A2. Table 2 provides an overview of the means and standard errors for the three dependent variables as a function of the main effects of the independent variables. In general, speakers included a redundant color attribute in 64% of the descriptions.

**Table 2:** The means and standard deviations errors for the three dependent variables of Experiment 2, as a function of all main effects analyzed.

		**Region of space**		**Type similarity**		**Color similarity**	
		**Same**	**Different**	**Same**	**Different**	**Same**	**Different**
Redundancy		0.65 (0.07)	0.63 (0.07)	0.69 (0.06)	0.59 (0.08)	0.60 (0.08)	0.68 (0.07)
Gaze dur. (ms)	Abs.	1825.5 (212.2)	437.8 (48.3)	1230.9 (132.0)	1032.4 (107.5)	1176.9 (120.8)	1086.5 (122.7)
	Rel.	0.43 (0.05)	0.09 (0.01)	0.27 (0.03)	0.25 (0.03)	0.27 (0.03)	0.25 (0.03)
No. of fixations	Abs.	2.07 (0.13)	1.49 (0.10)	1.92 (0.13)	1.64 (0.11)	1.84 (0.11)	1.72 (0.13)
	Rel.	0.17 (0.01)	0.11 (0.01)	0.15 (0.01)	0.14 (0.01)	0.15 (0.01)	0.14 (0.01)

#### Results for Redundant Color Use

**Figure 6:**
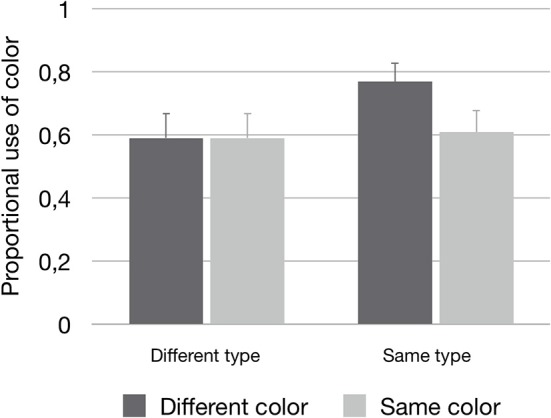
The proportional use of color (plus standard errors), depicted for the interaction effect between Type similarity and Color similarity.

The first ANOVA was performed to test if redundant color use was affected by the three manipulations of perceptual grouping. The first factor that was expected to affect redundant color use was region of space. However, there was no significant effect here [*F*1_(1, 29)_ = 0.39, n.s.; *F*2_(1, 24)_ = 0.17, n.s.; *minF*′_(1, 43)_ = 0.12, n.s.]: speakers used color equally often when the manipulated distractor was in the same or a different region of space as compared to the target (see Table 2 for the means). For the two manipulations of similarity, the analyses did show effects on the redundant use of color. In these cases, the main effects of type similarity [*F*1_(1, 29)_ = 8.21; *p* = 0.008; ${\eta_{\text{p}}}^{2}$ = 0.22; *F*2_(1, 24)_ = 15.17; *p* = 0.001; ${\eta_{\text{p}}}^{2}$ = 0.39; *minF*′_(1, 51)_ = 5.23; *p* = 0.03] and color similarity [*F*1_(1, 29)_ = 15.46; *p* < 0.001; ${\eta_{\text{p}}}^{2}$ = 0.35; *F*2_(1, 24)_ = 8.70; *p* = 0.007; ${\eta_{\text{p}}}^{2}$ = 0.27; *minF*′_(1, 46)_ = 5.57; *p* = 0.02] should be interpreted in the light of a significant interaction between type similarity and color similarity [*F*1_(1, 29)_ = 11.44; *p* = 0.002; ${\eta_{\text{p}}}^{2}$ = 0.28; *F*2_(1, 24)_ = 11.90; *p* < 0.002; ${\eta_{\text{p}}}^{2}$ = 0.33; *minF*′_(1, 53)_ = 5.83; *p* = 0.02]. The pattern for this interaction showed an increase in redundant color use when the manipulated distractor had the same type as the target, and a different color (*M* = 0.77, SE = 0.06). The other three cells were practically indistinguishable (same type – same color: *M* = 0.61, SE = 0.07; different type – same color: *M* = 0.59, SE = 0.08; different type – different color: *M* = 0.59, SE = 0.08). The pattern is depicted in Figure 6.

#### Results for Gaze Duration

The second ANOVA was run to analyse if the manipulations of grouping affected the total amount of time that speakers looked at the manipulated distractor. Firstly, there was a main effect of region of space on gaze duration, both for the absolute scores [*F*1_(1, 23)_ = 49.26; *p* < 0.001; ${\eta_{\text{p}}}^{2}$ = 0.68; *F*2_(1, 24)_ = 192.36; *p* < 0.001; ${\eta_{\text{p}}}^{2}$ = 0.89; *minF*′_(1, 34)_ = 39.22; *p* < 0.001] and the relative scores [*F*1_(1, 23)_ = 37.42; *p* < 0.001; ${\eta_{\text{p}}}^{2}$ = 0.62; *F*2_(1, 24)_ = 1293.20; *p* < 0.001; ${\eta_{\text{p}}}^{2}$ = 0.98; *minF*′_(1, 24)_ = 36.37; *p* < 0.001]. As can be seen in Table 2, this effect showed that the distractor object was looked at significantly longer when it was placed in the same rather than a different region of space than the target.

A similar effect was found for the manipulation of type similarity, although only for the F1 analysis on the absolute scores [*F*1_(1, 23)_ = 16.86; *p* < 0.001; ${\eta_{\text{p}}}^{2}$ = 0.42; *F*2_(1, 24)_ = 2.26, n.s.; *minF*′_(1, 30)_ = 1.99, n.s.], and not for the relative scores [*F*1_(1, 23)_ = 2.23, n.s.; *F*2_(1, 24)_ = .32, n.s.; *minF*′_(1, 31)_ = 0.28, n.s.]. The absolute scores suggested that distractors that shared their type with the target were looked at longer than distractors with a different type (see Table 2). The third factor, color similarity, did not affect gaze duration. Although the distractor was looked at slightly longer when it had the same rather than a different color than the target, this difference was not significant, neither for the absolute scores [*F*1_(1, 23)_ = 2.23, n.s.; *F*2_(1, 24)_ = 0.68, n.s.; *minF*′_(1, 37)_ = 0.52, n.s.], nor for the relative scores [*F*1_(1, 23)_ = 2.48, n.s.; *F*2_(1, 24)_ = 1.94, n.s.; *minF*′_(1, 47)_ = 1.09, n.s.]. The means can again be found in Table 2. Interaction effects were not found here.

#### Results for Number of Fixations

The third dependent variable was the number of fixations on the manipulated distractor. Again, there were main effects of region of space and type similarity, but not of color similarity. Firstly, as can be seen in Table 2, when the distractor was in the same region of space as the target object, participants looked at this object more often than when it occurred in a different region of space. This effect was significant for both the absolute scores [*F*1_(1, 23)_ = 74.66; *p* < 0.001; ${\eta_{\text{p}}}^{2}$ = 0.76; *F*2_(1, 24)_ = 24.92; *p* < 0.001; ${\eta_{\text{p}}}^{2}$ = 0.51; *minF*′_(1, 38)_ = 18.68; *p* < 0.001], and the relative scores [*F*1_(1, 23)_ = 75.32; *p* < 0.001; ${\eta_{\text{p}}}^{2}$ = 0.77; *F*2_(1, 24)_ = 127.24; *p* < 0.001; ${\eta_{\text{p}}}^{2}$ = 0.84; *minF*′_(1, 44)_ = 47.31; *p* < 0.001].

Secondly, the means for type similarity showed that distractors with the same type as the target object were fixated more often than distractors with a different type (see Table 2). However, this effect only reached significance for the absolute scores [*F*1_(1, 23)_ = 16.61; *p* < 0.001; ${\eta_{\text{p}}}^{2}$ = 0.42; *F*2_(1, 24)_ = 6.73; *p* = 0.02; ${\eta_{\text{p}}}^{2}$ = 0.22; *minF*′_(1, 40)_ = 4.79; *p* = 0.04], and not the relative ones [*F*1_(1, 23)_ = 1.31, n.s.; *F*2_(1, 24)_ = 0.44, n.s.; *minF*′_(1, 38)_ = 0.33, n.s.]. The effect of color similarity was again not significant. Thus, the distractor color did not affect the number of fixations, neither for the absolute scores [*F*1_(1, 23)_ = 2.07, n.s.; *F*2_(1, 24)_ = 1.19, n.s.; *minF*′_(1, 44)_ = 0.76, n.s.), nor for the relative scores [*F*1_(1, 23)_ = 2.06, n.s.; *F*2_(1, 24)_ = 1.90, n.s.; *minF*′_(1, 47)_ = 0.99; n.s.]. Interaction effects were not found either.

### Interim Discussion

The goal of Experiment 2 was to test how three manipulations of perceptual grouping guide speakers in determining the distractor set in a given visual scene, and how fixating (or ignoring) certain distractors leads to overspecification with color. While Experiment 1 only took offline measures of scene perception, the current experiment measured attention both in direct (eye tracking) and indirect (occurrence of overspecification) ways. There were three manipulations of perceptual grouping (i.e., common region of space, color similarity, and type similarity), all realized by varying the location and characteristics of one specific distractor object in the visual scenes that were presented to the participants.

The first manipulation made the manipulated distractor object appear either in the same or a different region of space as compared to the target referent. In Experiment 1, this manipulation led to a significant effect of region of space on overspecification, with more redundant color attributes in the “same group” condition rather than the “different group” condition. In this second experiment, this result could not be replicated: the proportions of overspecification were exactly the same in both conditions. However, both the analyses of the absolute and the relative scores showed effects of region of space in the eye-tracking data: when the distractor was in the same region as the target referent, it was viewed longer and more often than when it was in a different region. This way, region of space (Palmer, 1992) influences the extent to which certain distractors are considered in a reference production task. The question is why the patterns for common region of space that were observed in the eye-tracking data were not reflected in effects on overspecification with color, such as in Experiment 1. Given that one explanation may be related to the use of 3D scenes in the first experiment, a discussion of this question is provided in the General Discussion.

For type similarity, the effects on overspecification with color matched the observed pattern in the eye-tracking data, but only for the F1 analysis on the absolute scores. For color similarity, there was a significant interaction with type similarity in the speech data, with an increase of overspecification with color when the distractor had the same type as the target, and a different color. This interaction is a replication of Koolen et al. (2016). In the eye-tracking data, however, there were no significant effects or interactions with color similarity involved. Again, I come back to the effects of type and color similarity in the General Discussion, as well as to the link between scene perception and reference production.

## General Discussion

The two experiments described in this paper explored the impact of perceptual grouping and presentation mode (2D vs. 3D) on how speakers perceive distance between objects in a visual scene when referring to objects. In Experiment 1, the manipulation of presentation mode did not reveal reliable effects on redundant color use. Nonetheless, the analyses did show effects of region of space and type similarity on overspecification with color, implying that objects that share their region or type with the target are more likely to be considered a relevant distractor than objects for which this is not the case. In Experiment 2, some of these patterns were indeed reflected in speakers' eye movements, with a longer gaze duration and more fixations on distractors within the target's region of space, and, to some extent, with the target's type. Effects of the third manipulation of grouping, color similarity, were only present for overspecification with color, but not in the eye tracking data.

An interesting pattern in the current experiments was observed for one of the classical laws of perceptual grouping, as defined by Wertheimer (1923): similarity, in this case *type similarity*. In both experiments, type similarity significantly affected redundant color use, and the patterns observed in the eye tracking data were to some extent in line with these effects: they were only significant for the analyses on the absolute scores, but not the relative ones. Altogether, these findings provide tentative evidence for the close connection between visual scene perception and language production, in line with some previous work in this direction. For example, Davies and Kreysa (2017) showed that fixations on distractor objects before the onset of referring expressions make it less likely for speakers to produce underspecified expressions. Another recent paper by Elsner et al. (2018) looked into the relation between visual complexity of a scene, scan patterns, and speech onset times. In their experiment, participants produced descriptions of target objects in large grids of colored squares and circles. Central to their eye tracking analysis was scan distance: the total amount of distance that fixations traversed. Scan distance was taken as a measure of the amount of visual scanning that speakers have done at a specific point in time. In their research, Elsner et al. (2018) built on Gatt et al. (2017), who found that speech onset times get longer as there are more objects present in a domain, especially in cases where the target fails to pop out of the scene.

Although Elsner et al. (2018) used abstract scenes without systematic manipulations of grouping, their results can be linked to the findings for type similarity observed in the current paper. One of their main results was that speech onset times increase when a distractor is present that is visually similar to the target. Notably, this effect only held for trials where speakers' initial scan of the scene was quite extensive, and not for trials where speakers did not scan a considerable part of the scene before starting to speak. Because the objects in their scenes were physically too close to each other, Elsner et al. (2018) did not give calculations of fixations on specific distractors. However, their results still suggest that type similarity affects reference production: after a considerable scan of the scene, objects that are similar to the target are indeed likely to be considered a relevant distractor, which causes the reference production process to slow down. The effects of type similarity reported in the current study (at least the ones for overspecification with color) are in line with this suggestion, extending it to more photo-realistic scenes and a controlled physical distractor distance.

While the results for type similarity reported on here are consistent across the speech and eye tracking data, this is not the case for *color similarity*. For this factor, the speech data replicated the interaction that was found earlier by Koolen et al. (2016): the occurrence of overspecification increased when the manipulated distractor had a different color than the target, but only when it also had the same type. However, in the eye tracking data, there were no significant main effects or interactions with color similarity involved. One explanation could be that color differences are easily perceived, because they usually “pop out” of the scene (Treisman and Gelade, 1980), irrespective of domain size (Wolfe, 2012). For that reason, it may be that in the current experiments, there was no strict need for speakers to fixate distractors (repeatedly) in order to perceive the different colors; they may have already perceived the color difference without fixating any objects. Following this explanation, it is plausible to assume that color similarity did affect speakers during the reference production task, in terms of both overspecification and visual scene perception, but that for the latter, grouping strategies could not be caught by the eye tracker.

For the third manipulation of grouping applied here, *region of space*, there were effects in both the speech data (Experiment 1) and the eye tracking data (Experiment 2). The effects were in line with the hypotheses. The most important question that remains is why the region of space manipulation did not affect the occurrence of overspecification in the second experiment, while it did in the first. The explanation of this issue may lie in some practical differences between the two experiments. Firstly, Experiment 1 displayed the stimuli on a big television screen, while Experiment 2 used only 70% of a computer screen. Perhaps more important was that when looking at the means for the different conditions, the first experiment showed a convincing effect on overspecification in 3D visual scenes, but not so much in 2D scenes. In the light of that observation, it is not surprising that region of space did not affect overspecification in the second experiment, since only 2D scenes were applied there, due to restrictions of the eye tracking paradigm.

Although the explanation for the different patterns for the effect of region of space on overspecification may be found in presentation mode, the interaction between region of space and presentation mode in Experiment 1 was only significant by participants, and thus not fully reliable. The same goes for the main effect of presentation mode, which was only significant by items. Therefore, it is interesting to speculate about the extent to which presentation mode shapes the perceived distance between the objects in a scene.

In general, for presentation mode, it was expected that it is more difficult for speakers to accurately perceive the distance between objects in 2D rather than 3D scenes, since in 3D, they can rely on both monocular and binocular cues for depth perception (Loomis, 2001; McIntire et al., 2014). For the main effect of presentation mode, the difference between the means was in the expected direction, with higher proportions of overspecification in 2D than 3D. So why was this difference only significant by items? I conjecture that this is due to (too) much variation between individual speakers, following recent observations on speaker differences in related work (e.g., Hendriks, 2016; Elsner et al., 2018). For presentation mode, these differences would imply that binocular cues are more effective for one viewer than the other (Wilmer, 2008). For example, some speakers may be able to perceive depth and distance with monocular cues only, while others need a combination with binocular cues to do so.

Despite the assumed impact of individual variation, the pattern for the means of the interaction between presentation mode and region of space tells an alternative story. Although only significant by participants, this pattern suggests that region of space had the strongest impact in 3D, leading to a higher proportion of redundant color use in the same region condition than the different region condition, while this difference was practically absent in 2D. For one thing, the numerical difference between the conditions in 3D implies that the combination of monocular and binocular depth cues enhances the effect of region of space. However, as explained in the Discussion of Experiment 1, one would expect that in 3D, speakers are better at inferring that the physical distance between the target and the distractor in the two region of space conditions was in fact the same. One explanation for the unexpected pattern here could be that the impact of 3D presentation mode on distance and depth estimations depends on the way it is manipulated. In the current study, this was done horizontally, on the X-axis. It would be relevant to test if the effect of presentation mode is stronger when distance is manipulated along the depth (Z) axis, or along the X-axis and the Z-axis at the same time. In those cases, the effect of region of space could actually be stronger in 3D than 2D, instead of the other way around, since the presence of binocular cues would then pay off more convincingly.

On top of additional manipulations of distance in 3D, various alternative directions for future research are relevant to think about. Firstly, there is the effect of individual variation that can be studied systematically, for example by testing participants before the experiment on their ability to perceive depth and distance, and to see how this affects their perception of a scene, as well as reference production. Secondly, it may be relevant to test the effects of the current manipulations on variables such as the number of words that speakers use for their referring expression, or the occurrence of underspecification, especially in even more large-scale experiments. Furthermore, it would be interesting to replicate the current experiments in a Virtual Reality environment that facilitates the collection of eye movements. In such an environment, the effect of presentation mode, and its interactions with perceptual grouping, could be tested with a direct measure of visual attention. By using VR, the ecological validity of studies like the ones presented here could be further enhanced, while keeping maximum experimental control (Peeters, 2019). Many labs do not currently offer a VR environment with eye tracking facilities, but such facilities may become common in the (perhaps even near) future.

Although I am in favor of doing reference production research in 3D or VR settings, with a combination of offline and online measures of visual attention such as the ones applied in the current study, there is one potential drawback that should be acknowledged, namely the risk of under-powered studies. In the ideal scenario, plenty of participants are tested, who all produce multiple referring expressions. However, given that participants have to get accustomed to their task and the (lab/3D/VR) environment; too lengthy and repetitive experiments should be avoided; and data transcription and annotation is time-consuming, studies like the ones presented here often have lower statistical power than desired. For the current experiments, the number of data points that was used for the final analyses was indeed rather low. The result is a potentially lower power that may have repercussions for the interpretation of the findings. For example, one question is if the null results should be taken as evidence for no effect, or that the effect was too small to detect given the size of the data set. For the experiments presented here, the latter may be the case, also because the effects of the current manipulations are arguably rather small by nature (for example due to individual variation between speakers; see above). Thus, although the number of participants in the current experiments was comparable to related studies, and each condition always had four repeated items, aiming for more power could be taken as another reason to re-test the effects of grouping on reference production in follow-up studies.

Finally, it is relevant to discuss several methodological choices that were made in the two experiments. Firstly, arrows were used to indicate the target referent, which may have steered attention, and, in turn, participants' eye movements and tendency to overspecify. Although the use of arrows (or squares) to indicate the target is common practice in research on visually-grounded reference production (e.g., Van Gompel et al., 2019, among many others), it may be wise for follow-up experiments to cue the target before trial onset. Secondly, in these kinds of experiments, there is a potential risk of learning effects that may occur over the course of the experiment. For example, in the first experiment, participants may have learned that the left side of the visual scene was irrelevant to their task, and that they could thus ignore it. Furthermore, in both experiments, each set of objects was referred to repeatedly, with similar targets, which may have resulted in potential repetition effects. Although I do acknowledge the risk of these kinds of learning effects, there are various reasons to believe that they did not affect the results of the current experiments: the amount of filler trials was high, especially in Experiment 1; in both experiments, the size of the target referent was either big or small; in Experiment 1, the two trial orders that were applied did not affect overspecification with color; and in Experiment 2, the fact that half of the trials were mirrored did not affect the participants' scores on the dependent variable, nor interact with any of the independent variables that were manipulated. However, still, the different approaches with trial order and mirroring between the two experiments may cause the two experiments to be not directly comparable.

Again in terms of methodology, I would like to end with some reflection on the decision to use subsets of the data for the eye-tracking analyses in Experiment 2. For these subsets, only data was included for trials where the speaker fixated the AOI of the manipulated distractor at least once. One can argue that this approach is too strict, because speakers do not always scan the complete scene before producing a referring expression (e.g., Elsner et al., 2018). However, still, there were reasons to require at least one fixation on the distractor. Firstly, it was now likely that speakers were aware of the existence of the distractor, which is important if one wants to draw conclusions on effects of distractor characteristics. Secondly, it excluded measurement errors that occur when speakers changed their position in front of the eye-tracker.

## Conclusion

In two experiments, the current paper tested the impact of three manipulations of perceptual grouping, as well as presentation mode (2D vs. 3D) on how people perceive the distance between objects in a visual scene when referring to objects. The results showed an effect of region of space, type similarity, and color similarity on the redundant use of color, suggesting that some objects are more likely to be considered relevant distractors than others. In the second experiment, this suggestion was verified with a direct measure of visual attention, eye tracking, for which the analyses indeed showed convincing effects of region of space in the expected directions. Hypothesized effects of presentation mode (2D vs. 3D) were not convincingly borne out by the data.

## Data Availability Statement

The datasets generated for this study are available on request to the corresponding author.

## Ethics Statement

All participants signed a consent form, which was approved by the ethics committee of the Tilburg Center for Cognition and Communication (Tilburg University).

## Author Contributions

The author confirms being the sole contributor of this work and has approved it for publication.

### Conflict of Interest

The author declares that the research was conducted in the absence of any commercial or financial relationships that could be construed as a potential conflict of interest.

## Appendix

**Table A1:** Additional (non-significant) interaction effects for Experiment 1.

	***F*1**	***F2***	**min*F^**′**^***
Type × Region	*F*1_(1, 46)_ = 0.76; *p* = 0.39	*F*2_(1, 12)_ = 0.73; *p* = 0.41	*minF′*_(1, 37)_ = 0.37; *p* = 0.55
Type × Presentation mode	*F*1_(1, 46)_ = 3.51; *p* = 0.07	*F*2_(1, 12)_ = 3.25; *p* = 0.10	*minF′*_(1, 36)_ = 1.69; *p* = 0.20
Type × Region × Pres. mode	*F*1_(1, 46)_ = 0.02; *p* = 0.90	*F*2_(1, 12)_ = 0.01; *p* = 0.93	*minF′*_(1, 25)_ = 0.01; *p* = 0.94

**Table A2:** Additional (non-significant) interaction effects for Experiment 2.

			***F*1**	***F*2**	***minF*^**′**^**
Redundancy		Region × Type	*F*1_(1, 29)_ = 0.19; *p* = 0.67	*F*2_(1, 24)_ = 0.06; *p* = 0.81	*minF′*_(1, 38)_ = 0.05; *p* = 0.83
		Region × Color	*F*1_(1, 29)_ = 1.06; *p* = 0.31	*F*2_(1, 24)_ = 0.48; *p* = 0.49	*minF′*_(1, 43)_ = 0.33; *p* = 0.57
		Region × Type × Color	*F*1_(1, 29)_ = 0.00; *p* = 0.97	*F*2_(1, 24)_ = 0.00; *p* = 0.99	*minF′*_(1, 53)_ = 0.00; *p* = 1.00
Gaze dur. (ms)	Abs.	Region × Type	*F*1_(1, 29)_ = 0.03; *p* = 0.87	*F*2_(1, 24)_ = 0.34; *p* = 0.56	*minF′*_(1, 34)_ = 0.03; *p* = 0.87
		Region × Color	*F*1_(1, 29)_ = 0.31; *p* = 0.58	*F*2_(1, 24)_ = 0.49; *p* = 0.49	*minF′*_(1, 52)_ = 0.19; *p* = 0.67
		Region × Type × Color	*F*1_(1, 29)_ = 2.13; *p* = 0.16	*F*2_(1, 24)_ = 1.53; *p* = 0.23	*minF′*_(1, 50)_ = 0.89; *p* = 0.35
	Rel.	Region × Type	*F*1_(1, 29)_ = 0.65; *p* = 0.43	*F*2_(1, 24)_ = 3.73; *p* = 0.07	*minF′*_(1, 39)_ = 0.55; *p* = 0.46
		Region × Color	*F*1_(1, 29)_ = 0.73; *p* = 0.40	*F*2_(1, 24)_ = 0.08; *p* = 0.79	*minF′*_(1, 29)_ = 0.07; *p* = 0.79
		Region × Type × Color	*F*1_(1, 29)_ = 0.18; *p* = 0.68	*F*2_(1, 24)_ = 0.05; *p* = 0.83	*minF′*_(1, 37)_ = 0.04; *p* = 0.84
No. of fixations	Abs.	Region × Type	*F*1_(1, 29)_ = 2.80; *p* = 0.11	*F*2_(1, 24)_ = 2.62; *p* = 0.12	*minF′*_(1, 52)_ = 1.35; *p* = 0.25
		Region × Color	*F*1_(1, 29)_ = 0.02; *p* = 0.88	*F*2_(1, 24)_ = 0.03; *p* = 0.87	*minF′*_(1, 52)_ = 0.01; *p* = 0.91
		Region × Type × Color	*F*1_(1, 29)_ = 0.53; *p* = 0.47	*F*2_(1, 24)_ = 0.00; *p* = 0.95	*minF′*_(1, 24)_ = 0.00; *p* = 1.00
	Rel.	Region × Type	*F*1_(1, 29)_ = 0.83; *p* = 0.37	*F*2_(1, 24)_ = 0.00; *p* = 0.98	*minF′*_(1, 24)_ = 0.00; *p* = 1.00
		Region × Color	*F*1_(1, 29)_ = 0.06; *p* = 0.81	*F*2_(1, 24)_ = 0.12; *p* = 0.73	*minF′*_(1, 50)_ = 0.04; *p* = 0.84
		Region × Type × Color	*F*1_(1, 29)_ = 1.21; *p* = 0.28	*F*2_(1, 24)_ = 0.84; *p* = 0.37	*minF′*_(1, 49)_ = 0.50; *p* = 0.49
